# Myricitrin Delays *Caenorhabditis elegans* Aging: Association with DAF-16, SIR-2.1, and Potential Cholinergic Modulation

**DOI:** 10.3390/antiox15101230

**Published:** 2026-09-23

**Authors:** Lijun Yang, Jiuyi Li, Yihan Zhou, Huaying Xu, Yu Wang, Jiayuan Zhao, Yanan Wang

**Affiliations:** School of Life Sciences, Sichuan Normal University, Chengdu 610101, China; 20251501050@stu.sicnu.edu.cn (L.Y.); 20220019@sicnu.edu.cn (J.L.); 20221501024@stu.sicnu.edu.cn (Y.Z.); 20231501060@stu.sicnu.edu.cn (H.X.); 20010066@sicnu.edu.cn (Y.W.); jiayuanzhao@sicnu.edu.cn (J.Z.)

**Keywords:** aging, antioxidant, mitochondria, myricitrin, *Caenorhabditis elegans*

## Abstract

Background: Aging involves progressive functional decline driven by oxidative stress and mitochondrial dysfunction, and dietary flavonoids are promising aging interventions due to their multi-target regulatory pathways. Methods: This study systematically evaluated the anti-aging activity of myricitrin (MYR) and its underlying mechanisms using *Caenorhabditis elegans*, testing MYR at 0.25 mg/mL, employing a rotenone-induced model of mitochondrial oxidative stress, and performing reporter strain analyses, qRT-PCR, and molecular docking analysis with acetylcholinesterases (ACE-1/2). Results: MYR at 0.25 mg/mL significantly extended lifespan and improved locomotor performance without detectable toxicity regarding growth, feeding, or reproduction; reduced reactive oxygen species accumulation and restored antioxidant enzyme activities in the rotenone-induced model; was accompanied by the nuclear translocation of DAF-16 and the upregulation of associated downstream antioxidant genes (*sod-3*, *gst-4*); enhanced mitochondrial quality control by upregulating *sir-2.1* and modulating genes associated with the mitochondrial unfolded protein response (*atfs-1*, *hsp-6*) and mitochondrial dynamics (*fzo-1*, *drp-1*); and indicated a favorable interaction between MYR and acetylcholinesterases (ACE-1/2). Conclusions: MYR represents a promising candidate compound for mitigating aging-related phenotypes, with its beneficial effects linked to a DAF-16-associated antioxidant response and SIR-2.1-correlated mitochondrial quality control network.

## 1. Introduction

Population aging has become a major global health challenge. The number of individuals aged 65 years and older is projected to exceed 1.6 billion by 2050 [1]. Although life expectancy has increased substantially, gains in healthspan have been far more limited. As a result, age-related disorders are imposing an increasing burden on healthcare systems and society worldwide [2]. This gap between lifespan and healthspan has intensified interest in safe dietary or natural compounds that can delay functional decline and promote healthy aging.

Among the recognized hallmarks of aging, mitochondrial dysfunction is particularly important because it directly affects cellular energy metabolism, redox balance, and stress resistance [3,4]. Under physiological conditions, mitochondrial homeostasis is maintained by the mitochondrial quality control (MQC) system, which coordinates protein folding, organelle dynamics, biogenesis, and the removal of damaged mitochondrial components. As aging progresses, however, excessive reactive oxygen species (ROS) accumulation disrupts this balance. Oxidative stress damages mitochondrial macromolecules, while sustained mitochondrial injury further amplifies ROS production, creating a self-reinforcing cycle that accelerates functional decline [5]. This process is associated with reduced respiratory chain stability, impaired mitochondrial biogenesis, and compromised energy metabolism [6]. Targeting oxidative stress and mitochondrial quality control therefore represents a rational strategy for delaying aging and mitigating age-related deterioration [7].

Natural flavonoids, abundant in various fruits, vegetables, and beverages, have attracted considerable attention as potential anti-aging agents and functional dietary ingredients. This is because they often act on multiple molecular targets and exhibit favorable bioactivities across experimental systems [8]. Their protective effects are commonly linked to antioxidant activity, redox regulation, and preservation of mitochondrial function [9]. Myricitrin (MYR), a glycosylated derivative of myricetin, is a naturally occurring polyhydroxylated flavonoid widely distributed in edible plants, such as Chinese bayberry (*Myrica rubra*), teas, and various nutritional herbs. It possesses reported antioxidant, anti-inflammatory, neuroprotective, and cardioprotective properties [10,11,12]. Compared with its aglycone, glycosylation improves its water solubility, a critical factor for the bioavailability and physiological efficacy of food-derived bioactives. Previous studies have shown that MYR can alleviate metabolic stress and reduce intracellular ROS accumulation [13,14]. However, current evidence remains fragmented. In particular, whether MYR delays aging through coordinated regulation of antioxidant defense and mitochondrial quality control has not been clearly established. Moreover, its potential connection with cholinergic signaling, a pathway closely related to neuromuscular function and age-associated physiological decline, has rarely been explored.

*Caenorhabditis elegans* is a well-established model for aging research because of its short lifespan, genetic tractability, and highly conserved longevity-associated pathways, including insulin/IGF-1 signaling, SIR-2.1/SIRT1, and AMPK [6,15]. Under standard laboratory conditions (20 °C), *C. elegans* exhibits a rapid life cycle, developing from eggs through four distinct larval stages (L1 to L4) to egg-laying adults in approximately 3.5 days, with a mean adult lifespan of 18–20 days [16]. It is also particularly suitable for evaluating natural compounds that target mitochondrial homeostasis, oxidative stress resistance, and locomotor function in vivo. In our experimental design, the wild-type N2 strain was used to evaluate the regulatory effects of MYR on fundamental lifespan and physiological phenotypes such as motility. Concurrently, multiple transgenic reporter strains enable direct visualization of pathway activity, making this model especially useful for linking phenotypic outcomes to real-time molecular mechanisms. In addition, transgenic reporter strains enable direct visualization of pathway activity, making this model especially useful for linking phenotypic outcomes to molecular mechanisms. These advantages provide an efficient platform for identifying food-derived or plant-derived bioactives with genuine healthy-aging potential rather than merely lifespan-extending effects.

In this study, we used *C. elegans* to systematically evaluate the anti-aging activity of MYR and to define its mechanism of action from a multi-target perspective. We examined its effects on lifespan, locomotion, growth, feeding, and reproduction, and further assessed its protective capacity in a rotenone-induced oxidative stress model. By integrating fluorescent reporter analysis, mitochondrial functional assessment, qRT-PCR, and network pharmacology, we investigated whether MYR acts through coordinated regulation of DAF-16-associated antioxidant response, SIR-2.1-related mitochondrial quality control, and potential cholinergic modulation. This design highlights the main innovation of the study: not simply showing that MYR has antioxidant activity, but demonstrating that its anti-aging effects are associated with an integrated regulatory network that links DAF-16 activation, stress resistance, mitochondrial homeostasis, and functional maintenance. Such evidence may strengthen the rationale for developing MYR as a candidate functional ingredient for healthy aging.

## 2. Materials and Methods

### 2.1. Materials

Myricitrin (MYR; Cat. No. S24986-1g) was purchased from Yuanye Biotechnology Co., Ltd. (Shanghai, China). Rotenone (Cat. No. R817233-5 g) was obtained from Aladdin Reagent Company (Shanghai, China). The reactive oxygen species (ROS) detection kit (Cat. No. R22380) was purchased from Yuanye Biotechnology Co., Ltd. (Shanghai, China). The bicinchoninic acid (BCA) protein assay kit (Cat. No. P0012) was obtained from Beyotime Biotechnology (Shanghai, China). The glutathione peroxidase (GPx) assay kit (Cat. No. A005-1-2) was purchased from Nanjing Jiancheng Bioengineering Institute (Nanjing, China). The superoxide dismutase (SOD) assay kit (Lot No. 20241119) and the adenosine triphosphate (ATP) assay kit (Lot No. 20250110) were obtained from Shanghai Jianglai Biotechnology Co., Ltd. (Shanghai, China). The JC-1 mitochondrial membrane potential assay kit (Cat. No. CC1520) was purchased from Beijing Jinclone Biotechnology Co., Ltd. (Beijing, China). Kits for RNA extraction, reverse transcription, and quantitative polymerase chain reaction (qPCR) were obtained from Chengdu Foregene Biotechnology Co., Ltd. (Chengdu, China). Stock solutions of MYR (62.5, 125, 250, and 500 mg/mL) were prepared in dimethyl sulfoxide (DMSO) and stored at −20 °C for no more than two months. Working solutions containing 0.1% (*v*/*v*) DMSO as the vehicle control were freshly diluted immediately prior to treatment and maintained at 4 °C for up to one month. Nematode Growth Medium (NGM) and standard buffers were prepared following established protocols [17].

### 2.2. In Vitro Antioxidant Activity of Myricitrin

#### 2.2.1. 2,2-Diphenyl-1-picrylhydrazyl (DPPH) Radical Scavenging Assay

DPPH radical scavenging activity was measured as described by Xu et al. [18] with minor modifications. MYR was dissolved in dimethyl sulfoxide (DMSO) to final concentrations of 0, 0.0625, 0.125, 0.25, and 0.5 mg/mL (corresponding to 0, 134.6, 269.2, 538.3, and 1076.7 µM, respectively). Briefly, 630 μL of 0.2 mM DPPH solution was mixed with 70 μL of MYR solution in a 1.5 mL microcentrifuge tube. After vortexing, the mixture was incubated in a 37 °C water bath for 30 min. Absorbance was then measured at 515 nm using a microplate reader. L-ascorbic acid (AA) at the same concentrations (0, 0.0625, 0.125, 0.25, and 0.5 mg/mL, corresponding to 0, 0.35, 0.71, 1.42, and 2.84 mM, respectively) served as the positive control. The DPPH radical scavenging rate was calculated as follows:

Scavenging rate (%) = [1 − (*A*_1_ − *A*_0_)/*A*_2_] × 100%

where *A*_1_ is the absorbance of the reaction mixture containing MYR or AA, *A*_0_ is the absorbance of the sample solution (MYR or AA) without DPPH reagent, and *A*_2_ is the absorbance of the control.

#### 2.2.2. 2,2′-Azino-bis(3-ethylbenzothiazoline-6-sulfonic acid) (ABTS^•+^) Radical Scavenging Assay

The ABTS^•+^ scavenging activity was determined according to a previously reported method [18] with slight modifications. A 7 mM ABTS solution was mixed with 4.9 mM potassium persulfate at a 1:1 (*v*/*v*) ratio and incubated in the dark at room temperature for 16 h to generate the ABTS^•+^ stock solution. The resulting solution was diluted with phosphate buffer (pH 7.4) until the absorbance at 734 nm reached approximately 0.7. Next, 0.1 mL of MYR solution at different concentrations (0, 0.0625, 0.125, 0.25, and 0.5 mg/mL) was mixed with 3.9 mL of ABTS^•+^ solution. After incubation for 6 min, absorbance was measured at 734 nm. AA at the same concentrations was used as the positive control. The ABTS^•+^ scavenging rate was calculated as follows:

Scavenging rate (%) = (1 − *A*_1_/*A*_2_) × 100%

where *A*_1_ is the absorbance of the reaction mixture containing MYR or AA, and *A*_2_ is the absorbance of the control.

#### 2.2.3. Hydroxyl Radical (·OH) Scavenging Assay

Hydroxyl radical scavenging activity was evaluated using a Fenton reaction system, as described by Xu et al. [18] with minor modifications. Briefly, 200 μL of 9 mM FeSO_4_ was mixed with 200 μL of 10 mM H_2_O_2_ in a 2 mL tube to generate hydroxyl radicals. Then, 200 μL of MYR solution at different concentrations was added. After incubation for 10 min, 200 μL of 9 mM salicylic acid was added, and the mixture was further incubated in a 37 °C water bath for 30 min. Absorbance was measured at 510 nm. AA at the same concentrations served as the positive control. Hydroxyl radical scavenging activity was calculated as follows:

Scavenging activity (%) = [1 − (*A*_1_ − *A*_0_)/*A*_2_] × 100%

where *A*_1_ is the absorbance of the reaction mixture containing MYR or AA, *A*_0_ is the absorbance of the sample solution without Fenton reagents, and *A*_2_ is the absorbance of the control.

### 2.3. C. elegans Strains and Experimental Design

The *C. elegans* strains used in this study and their sources are listed in Table S1. All strains were maintained at 20 °C on nematode growth medium (NGM) plates seeded with *Escherichia coli* OP50. Briefly, *E. coli* OP50 was cultured overnight in LB liquid medium at 37 °C with shaking (120 rpm) until an optical density at 600 nm (OD_600_) of 0.3–0.6 was reached. For plate preparation, 100 µL of this OP50 bacterial suspension was seeded onto each NGM plate and allowed to dry to form a lawn. Synchronized populations were generated by bleach synchronization. Eggs were transferred to fresh OP50-seeded NGM plates and cultured to the L4 stage. For drug treatment, MYR was added to fresh NGM plates at final concentrations of 0, 0.0625, 0.125, 0.25, and 0.5 mg/mL. Synchronized L4-stage N2 worms were then transferred to MYR-containing plates for subsequent assays.

### 2.4. Assessment of C. elegans Physiological Parameters

#### 2.4.1. Lifespan Assay

For lifespan analysis, synchronized L4-stage N2 worms (*n* = 50 per group) were transferred daily to fresh NGM plates containing the indicated concentrations of MYR and maintained at 20 °C. The first day of transfer was defined as day 0 of adulthood. Survival was scored every 24 h until all worms had died.

#### 2.4.2. Pharyngeal Pumping Rate and Body Size

Synchronized L4-stage N2 worms were maintained on NGM plates containing the indicated concentrations of MYR for 4 days at 20 °C. For pharyngeal pumping analysis, treated worms were transferred to a 2% agarose pad on a microscope slide containing a small amount of OP50. Pumping rate was recorded as the number of pharyngeal contractions during an interval of 1 min under a stereomicroscope (*n* = 6 per group). For body size measurement, six worms per group were randomly selected, anesthetized with 1% sodium azide, mounted on 2% agarose pads, and imaged under a microscope to determine body length and width.

#### 2.4.3. Locomotion Assay

Synchronized L4-stage N2 worms were maintained on NGM plates containing the indicated concentrations of MYR for 4 days at 20 °C. After treatment, six worms per group were randomly selected and individually transferred to wells of a 24-well plate containing 1 mL of M9 buffer per well. Thrashing frequency was counted for 1 min under a stereomicroscope. One thrash was defined as a complete bend of the body from one side to the other.

#### 2.4.4. Brood Size Assay

Individual synchronized L4-stage N2 worms were transferred to separate NGM plates containing the indicated concentrations of MYR, with at least three replicate plates for each treatment. Worms were moved to fresh plates every 24 h for 4 consecutive days. The plates containing eggs were maintained at 20 °C, and the number of hatched L1 larvae was counted to determine the number of viable progeny.

### 2.5. Oxidative Stress Resistance Assay

Synchronized L4-stage N2 worms were pretreated on NGM plates containing the indicated concentrations of MYR for 4 days at 20 °C. After pretreatment, 15 worms from each group were randomly selected and transferred to NGM plates containing 1.5% H_2_O_2_ to induce acute oxidative stress. Survival was recorded at 1 h intervals until all worms had died. Animals that failed to respond to gentle stimulation of the head with a worm pick were scored as dead.

### 2.6. Establishment of a Rotenone-Induced Oxidative Stress Model in C. elegans

A 100 mM rotenone (RO) stock solution was prepared in dimethyl sulfoxide (DMSO) and serially diluted with K medium to final concentrations of 5, 10, 20, 40, and 80 μM. Synchronized L4-stage N2 worms were first cultured on standard NGM plates at 20 °C for 4 days to obtain young adults. For each concentration, 30 young adult worms were randomly transferred to plates containing the corresponding RO solution. After exposure for 36 h at 20 °C, survival was assessed and the survival rate was calculated. Based on these results, 20 μM RO, which reduced survival to below 50%, was selected for subsequent oxidative stress experiments.

For model establishment, worms were divided into three groups: Control, Model, and MYR treatment. Worms in the Control and Model groups were maintained on NGM plates without MYR, whereas those in the MYR treatment group were maintained on NGM plates supplemented with 0.25 mg/mL MYR. All groups were cultured at 20 °C for 4 days. To ensure a consistent supply of food and compound, worms were transferred to freshly prepared plates with the corresponding treatment on day 2. After 4 days, worms from all groups were collected and washed three times with M9 buffer in 1.5 mL microcentrifuge tubes to remove surface bacteria. For oxidative challenge, worms in the Control group were resuspended in 1 mL M9 buffer, whereas worms in the Model and MYR treatment groups were resuspended in 1 mL of 20 μM RO solution. Oxidative stress was induced by incubating the worms for 14 h at 20 °C before further analysis.

### 2.7. Assays for Reactive Oxygen Species and Antioxidant Responses

#### 2.7.1. Intracellular Reactive Oxygen Species Assay

Intracellular reactive oxygen species (ROS) levels were measured using the fluorescent probe 2′,7′-dichlorodihydrofluorescein diacetate (DCFH-DA). Worms were treated as described in Section 2.6. After treatment, worms were exposed to 20 μM RO for 3 h to induce oxidative stress. They were then collected in 1.5 mL microcentrifuge tubes and washed three times with M9 buffer to remove residual RO. Next, the worms were incubated with DCFH-DA at a final concentration of 100 μM in the dark at 20 °C for 2 h. For imaging, worms were immobilized on glass slides with 50 μL of 1% sodium azide (NaN_3_). Fluorescence images were acquired using a fluorescence microscope at excitation/emission wavelengths of 485/530 nm. At least six worms were imaged per group. Fluorescence intensity was quantified using ImageJ software (version 1.53k; National Institutes of Health, Bethesda, MD, USA).

#### 2.7.2. Glutathione Peroxidase and Superoxide Dismutase Activity Assays

After treatment as described in Section 2.6, worms were collected and washed three times with M9 buffer to remove adherent *E. coli* OP50. Samples were then homogenized in lysis buffer on ice using a cell disruptor (XM-900T; Xiaomei Ultrasonic Instrument (Kunshan) Co., Ltd., Kunshan, China; 720 W, 15 min). The homogenate was centrifuged at 4000 rpm for 20 min at 4 °C, and the supernatant was collected for analysis. Glutathione peroxidase (GSH-Px) and superoxide dismutase (SOD) activities were measured using commercial assay kits according to the manufacturers’ instructions. Total protein concentration was determined in parallel using a BCA protein assay kit for normalization.

#### 2.7.3. GST-4::GFP and SOD-3::GFP Fluorescence Assays

The transgenic strains CL2166 *{dvIs19 [(pAF15)gst-4p::GFP::NLS] III}* and CF1553 *{muIs84 [(pAD76) sod-3p::GFP + rol-6(su1006)]}* were used to monitor GST-4::GFP and SOD-3::GFP fluorescence, respectively. Worms were treated as described in Section 2.6. Six worms per group were randomly selected, anesthetized on glass slides with 50 μL of 1% sodium azide, and imaged immediately. Fluorescence images were captured using a fluorescence microscope at excitation/emission wavelengths of 485/530 nm. Fluorescence intensity was quantified using ImageJ software (National Institutes of Health, Bethesda, MD, USA).

### 2.8. Assay for DAF-16 and SKN-1 Nuclear Localization

The nuclear localization and expression of DAF-16 and SKN-1 proteins were assessed using the transgenic *C. elegans* strains TJ356 *{zIs356 [daf-16p::daf-16a/b::GFP + rol-6(su1006)]}* and LD1 *{ldIs7 [skn-1b/c::GFP + rol-6(su1006)]}*, respectively. Worms were treated as described in Section 2.6. Six worms from each group were randomly selected, anesthetized on glass slides with 50 μL of 1% sodium azide, and immediately observed under a fluorescence microscope. Images were acquired at excitation/emission wavelengths of 485/530 nm. Fluorescence intensity and subcellular localization were analyzed using ImageJ software (National Institutes of Health, Bethesda, MD, USA).

### 2.9. Mitochondrial Structure and Content

#### 2.9.1. Analysis of Mitochondrial Ultrastructure

Worms were treated as described in Section 2.6. After treatment, animals were collected into 1.5 mL microcentrifuge tubes and fixed in electron microscopy fixative at 4 °C for 12 h. Samples were then washed three times with 0.1 M phosphate buffer (pH 7.4) for 15 min each. All subsequent procedures, including post-fixation, dehydration, infiltration, embedding, sectioning, staining, and transmission electron microscopy (TEM) observation, were performed by Xingjin’ao Biotechnology Co., Ltd, Chengdu, China. At least six representative images showing mitochondrial ultrastructure were obtained for each group.

#### 2.9.2. Quantification of Mitochondrial Content

The transgenic strain PD4251 *{ccIs4251 [(pSAK2) myo-3p::GFP::LacZ::NLS + (pSAK4) myo-3p::mitochondrial GFP + dpy-20(+)]I}* was used to assess mitochondrial content in body wall muscle cells. Worms were treated as described in Section 2.6. After treatment, six worms per group were randomly selected, anesthetized on glass slides with 50 μL of 1% sodium azide, and imaged under a fluorescence microscope using GFP settings (excitation 485 nm, emission 530 nm). Fluorescence intensity was quantified using ImageJ software (National Institutes of Health, Bethesda, MD, USA).

### 2.10. Assessment of Mitochondrial Function

#### 2.10.1. ATP Content Assay

Worms were treated as described in Section 2.6. After treatment, animals were collected into 1.5 mL microcentrifuge tubes and washed three times with M9 buffer to remove residual *E. coli* OP50 from the body surface. Extraction buffer was then added, and the samples were homogenized on ice using a cell disruptor at 80% power for 15 min. The lysate was centrifuged at 12,000 rpm for 10 min at 4 °C, and the supernatant was collected and kept on ice for immediate analysis. A 250 μL aliquot of the supernatant was transferred to a new 1.5 mL tube, and the pH was adjusted to neutrality by adding a predetermined volume of extraction buffer B. ATP content was then measured using a commercial assay kit according to the manufacturer’s instructions.

#### 2.10.2. Mitochondrial Membrane Potential Assay

Mitochondrial membrane potential (ΔΨm) was assessed using the potentiometric fluorescent dye JC-1. In mitochondria with a high ΔΨm, JC-1 forms J-aggregates that emit red fluorescence, whereas under low ΔΨm conditions, it remains in the monomeric form and emits green fluorescence. The ratio of red to green fluorescence was therefore used as an indicator of ΔΨm. Worms were treated as described in Section 2.6. After treatment, animals were collected into 1.5 mL microcentrifuge tubes and washed three times with M9 buffer to remove residual *E. coli* OP50. The worms were then incubated with 1 mL of freshly prepared JC-1 working solution at 37 °C for 20 min. The working solution was prepared by mixing 50 μL of 200× JC-1 stock solution with 8 mL sterile ddH_2_O and 2 mL of 5× JC-1 staining buffer. A 1× JC-1 wash buffer was prepared in parallel. After incubation, excess dye was removed by washing the worms twice with the 1× JC-1 wash buffer. Six stained worms from each group were randomly selected, immobilized on glass slides with 50 μL of 1% sodium azide, and imaged under a fluorescence microscope using the red and green channels. The red-to-green fluorescence intensity ratio was quantified using ImageJ software (version 1.53k; National Institutes of Health, Bethesda, MD, USA).

#### 2.10.3. Assessment of Mitochondrial Respiratory Chain Complex Activities

The activities of mitochondrial respiratory chain complexes I-V were assessed using the transgenic *C. elegans* strain CB7272 *{mIs12 [myo-2p::GFP + pes-10p::GFP + F22B7.9p::GFP] II; frIs7 [nlp-29p::GFP + col-12p::DsRed] IV; uIs69 [pCFJ90(myo-2p::mCherry) + unc-119p::sid-1] V}*. Worms were treated as described in Section 2.6. After treatment, animals were collected into 1.5 mL microcentrifuge tubes and washed three times with M9 buffer to remove residual *E. coli* OP50. Six worms from each group were randomly selected, anesthetized on glass slides with 50 μL of 1% sodium azide, and imaged under a fluorescence microscope. Relative fluorescence intensity was quantified using ImageJ software (National Institutes of Health, Bethesda, MD, USA).

#### 2.10.4. Assessment of HSP-6 Expression

Expression of the mitochondrial unfolded protein response (UPR^mt^) chaperone HSP-6 was evaluated using the transgenic strain SJ4100 *{zcIs13 [hsp-6p::GFP + lin-15(+)]}*. Worms were treated as described in Section 2.6. After treatment, animals were collected into 1.5 mL microcentrifuge tubes and washed three times with M9 buffer to remove residual *E. coli* OP50. Six worms from each group were randomly selected, anesthetized on glass slides with 50 μL of 1% sodium azide, and imaged under a fluorescence microscope. Fluorescence intensity was quantified using ImageJ software (National Institutes of Health, Bethesda, MD, USA).

### 2.11. Quantitative Real-Time PCR (qRT-PCR)

Worms were treated as described in Section 2.6. After treatment, animals were collected into RNase-free microcentrifuge tubes and lysed in the appropriate lysis buffer. Total RNA was extracted using a commercial RNA isolation kit according to the manufacturer’s instructions. First-strand cDNA was synthesized from equal amounts of total RNA using a reverse transcription kit. Quantitative real-time PCR was performed using SYBR Green chemistry on a QuantStudio system, with *act-1* used as the internal reference gene. Gene-specific primers were designed using the web-based Primer-BLAST from NCBI website and synthesized commercially by Tsingke Biotechnology (Chengdu, China). All primer sequences are listed in Table S2.

### 2.12. Network Pharmacology and Molecular Docking

Potential anti-aging targets of myricitrin were identified using an integrated strategy that combined network pharmacology with molecular docking. Candidate myricitrin targets were retrieved from three databases: HERB 2.0, SwissTargetPrediction, and TCMSP. Due to the limited annotation of aging-related targets specifically for *C. elegans,* Aging-related human genes were collected from DrugBank, GeneCards, MalaCards, OMIM, and OpenTargets to provide a robust dataset for initial screening. The overlap between predicted myricitrin targets and aging-related genes was visualized using a Venn diagram generated with the R package VennDiagram (version 1.8.2). Overlapping targets were then used to construct a protein–protein interaction (PPI) network with the STRING database. The resulting network was imported into Cytoscape (version 3.10.4) for topological analysis. Key network parameters were calculated to identify hub nodes, which were regarded as the core candidate anti-aging targets of myricitrin.

Following the established cross-species network pharmacology approach [6], human aging-related targets were mapped to their orthologs in the *C. elegans* model. Specifically, screening was performed based on the built-in orthology table (Nematode orthologs) in WormBase, and the orthologous gene of human acetylcholinesterase (AChE) in *C. elegans* is *ace-1* [19]. Additionally, the protein encoded by the *C. elegans ace-2* gene is functionally homologous to human AChE [20,21]. For molecular docking, the structures of these homologous *C. elegans* proteins, ACE-1 (UniProt ID: P38433) and ACE-2 (UniProt ID: G5EDV9), were obtained from the UniProt database. The three-dimensional structure of myricitrin was retrieved from PubChem. Protein structures were preprocessed and converted to PDBQT format before docking. Docking simulations were performed using the AutoDock suite (version 1.5.7), with at least 20 independent runs conducted for each target. Binding energies generated by the AutoDock algorithm were used to evaluate binding affinity. Docking poses were visualized and analyzed using PyMOL (version 3.1.6.1).

### 2.13. Statistical Analysis

All experiments were performed with three independent biological replicates. Statistical analyses were conducted using SPSS software (version 25.0; SPSS Inc., Chicago, IL, USA). Differences among groups were evaluated by one-way analysis of variance (ANOVA), followed by Tukey’s honestly significant difference (HSD) post hoc test. Data are presented as mean ± SD. A value of *p* < 0.05 was considered statistically significant. Lifespan data were analyzed using the Kaplan–Meier method, and the Log-rank (Mantel–Cox) test was applied to determine the statistical significance of differences in survival curves between groups. Relative gene expression in qRT-PCR assays was calculated using the 2^−ΔΔCT^ method. GraphPad Prism 10 (GraphPad Software, San Diego, CA, USA) and Microsoft Excel 2021(Microsoft Corporation, Redmond, WA, USA) were used for statistical analysis and figure preparation.

## 3. Results

### 3.1. MYR Extends Lifespan and Improves Motility Without Compromising Basic Physiology

Myricitrin (MYR) extended lifespan in *C. elegans* over the tested range of 0.0625–0.5 mg/mL, with the strongest effect at 0.25 mg/mL (Figure 1A; Table S3). At this concentration, mean lifespan increased by 27.57% (*p* < 0.05, Log-rank test) and maximum lifespan by 14.29% relative to the control. MYR also alleviated the age-related decline in locomotion, as shown by a 12.08% increase in thrashing frequency at 0.25 mg/mL (Figure 1B, *p* < 0.05). In contrast, MYR did not significantly affect body length, body width, or brood size within the tested concentration range (Figure 1C,D,F and Figure S1). Pharyngeal pumping remained unchanged at 0.0625–0.25 mg/mL and increased only at 0.5 mg/mL (Figure 1E). These results identified 0.25 mg/mL as the optimal dose and indicate that MYR promotes healthy aging without evident developmental or reproductive cost.

### 3.2. MYR Exhibits Antioxidant Activity In Vitro and Improves Oxidative Stress Resistance In Vivo

MYR showed concentration-dependent scavenging activity against DPPH, ABTS^·+^, and hydroxyl radicals, reaching 91.73%, 92.69%, and 37.88%, respectively, at 0.5 mg/mL (Figure 2A–C). In the H_2_O_2_-induced oxidative stress model, MYR significantly improved survival (Figure 2D; Table S4). Again, 0.25 mg/mL was the most effective dose, extending maximum survival from 6 h to 13 h and increasing mean survival by 30.63% (*p* < 0.05, Log-rank test). Based on these results, 0.25 mg/mL MYR was used for subsequent mechanistic analyses.

### 3.3. MYR Attenuates Rotenone-Induced Oxidative Damage Through DAF-16-Associated Antioxidant Activation

A rotenone (RO)-induced oxidative injury model was established using 20 μM RO, which reduced worm survival to below 50% (Figure S2). Under these conditions, RO increased intracellular ROS by 47.73%, whereas MYR reduced ROS accumulation by 22.98% relative to the model group (Figure 3A,D, *p* < 0.05). RO also suppressed GST-4 and SOD-3 reporter fluorescence as well as GSH-Px and SOD activities, while MYR significantly restored all four antioxidant readouts (Figure 3B–H).

qRT-PCR further showed that RO downregulated *daf-16*, *sod-3*, *gst-4*, and *ctl-1*, whereas MYR significantly increased their expression (Figure 3I). In the TJ356 strain, MYR markedly promoted DAF-16 nuclear translocation, increasing the nuclear localization ratio by 176.92% relative to the RO group (Figure 4A,C *p* < 0.001). By contrast, MYR did not significantly reverse the RO-induced decrease in SKN-1::GFP fluorescence (Figure 4B,D). These data indicate that MYR mitigates oxidative damage primarily through a DAF-16-associated antioxidant response.

### 3.4. MYR Preserves Mitochondrial Structure and Content Under Rotenone Stress

Transmission electron microscopy showed that control worms maintained intact mitochondrial ultrastructure with dense cristae, whereas RO caused marked mitochondrial swelling and cristae disruption (Figure 5A). MYR substantially alleviated these structural abnormalities. Consistent with this observation, RO reduced mitochondrial GFP intensity in the PD4251 strain by 41.66%, while MYR increased the signal by 46.39% relative to the model group (Figure 5B,C, *p* < 0.001). These findings show that MYR protects mitochondrial integrity and limits mitochondrial loss during oxidative stress.

### 3.5. MYR Restores Mitochondrial Function and Selectively Modulates Mitochondrial Quality Control Pathways

In the CB7272 reporter strain, RO selectively reduced the signal corresponding to respiratory chain complexes I–III, whereas complexes IV–V were not significantly affected (Figure 6A,B,D,E). MYR restored the complexes I–III signal to approximately control levels. RO also suppressed HSP-6::GFP fluorescence, mitochondrial membrane potential, and ATP content, while MYR significantly reversed all three changes (Figure 6C,F–H). Together, these results indicate that MYR alleviates rotenone-induced mitochondrial dysfunction at both structural and functional levels.

At the transcriptional level, MYR significantly increased the expression of *fzo-1*, *atfs-1*, *sir-2.1*, and *hsp-6*, while decreasing *drp-1* expression (Figure 6I). In contrast, eat-3, pink-1, and lonp-1 were unchanged. This pattern suggests that MYR does not broadly activate all mitochondrial surveillance pathways, but instead selectively promotes SIR-2.1-associated mitochondrial quality control, UPR^mt^ signaling, and mitochondrial maintenance.

### 3.6. Computational Analyses Identify Potential Cholinergic-Related Targets of MYR

Network pharmacology identified overlapping targets between predicted MYR-binding proteins and aging-related genes, with acetylcholinesterase (ACHE) emerging as a prominent hub in the protein–protein interaction network (Figure 7A–C; Table S5). Molecular docking further predicted favorable binding of MYR to the *C. elegans* acetylcholinesterase homologs ACE-1 and ACE-2, with docking energies of −9.3 and −11.0 kcal/mol, respectively (Figure 7D,E). Although these results are computational, they support the possibility that cholinergic modulation contributes to the multi-target anti-aging action of MYR.

## 4. Discussion

Natural flavonoids are increasingly recognized as promising candidates for healthy-aging intervention because they often act on multiple, interconnected pathways involved in redox control, mitochondrial function, and stress adaptation [9]. As members of this family, myricetin [22] and its dihydro derivative, dihydromyricetin (DHM) [23], have demonstrated anti-aging potential. *Caenorhabditis elegans* is one of the most widely used model organisms in aging research, owing to its short lifespan, genetic tractability, and well-characterized age-dependent physiological decline [24]. In the present study, we examined myricitrin (MYR), a 3-O-rhamnoside derivative of myricetin, and found that it extended lifespan and improved locomotor performance in *C. elegans* without evident effects on growth, reproduction, or feeding at the effective concentration.

Interestingly, a higher concentration (0.5 mg/mL) yielded an attenuated longevity response compared to 0.25 mg/mL. This bell-shaped dose–response pattern is characteristic of a hormetic effect, a phenomenon frequently observed with dietary flavonoids [25]. Mechanistically, while moderate doses of flavonoids promote healthspan by upregulating endogenous cytoprotective pathways, higher doses may trigger mild pro-oxidant activity or intracellular redox alterations that offset these beneficial effects [25]. The concentration of 0.25 mg/mL represents a relatively high working dose, suggesting weak intrinsic potency of MYR within this model system. Consistent with most flavonoid glycosides, MYR exhibits poor oral absorption and rapid metabolic clearance in higher organisms, with limitations in pharmacokinetics and bioavailability [26]. Nevertheless, the high-dose requirement observed in the nematode model could be addressed by formulation strategies; novel delivery systems including nanoformulations and lipid-based nanocarriers have been validated to enhance the stability and in vivo bioavailability of dietary flavonoids [27,28,29]. Still, this threshold effect supports 0.25 mg/mL as the optimal physiological dose in our experimental setup to confer healthspan benefits without inducing secondary stress. Crucially, lifespan extension in *C. elegans* is not always accompanied by preserved physiological function [6]. Our data therefore support MYR as a compound that improves functional aging, rather than merely prolonging survival.

Oxidative stress is a well-established, critical driver of the aging process [30]. Flavonoids possess intrinsic free-radical scavenging capacity owing to their phenolic hydroxyl groups [31]. In our study, MYR confers biologically meaningful antioxidant protection, showing clear free-radical scavenging activity in vitro and improving survival under oxidative challenge in vivo. In a rotenone-induced stress model, MYR reduced intracellular ROS accumulation—an oxidative-injury modality linked to ferroptotic cell death [32]—and restored antioxidant readouts, including GST-4 and SOD-3 reporter signals, as well as GSH-Px and SOD activities. Mechanistically, MYR enhanced DAF-16 nuclear translocation and upregulated *daf-16* and its downstream antioxidant targets, whereas *skn-1* expression and SKN-1 reporter activity were not significantly altered, consistent with the pattern described by Büchter et al. [25]. While previous studies demonstrated that lifespan extension induced by the aglycone myricetin strictly requires DAF-16 [25] and that the longevity effects of its methylated derivatives are also largely dependent on this transcription factor [33], further functional loss-of-function genetic assays using *daf-16* mutants or RNAi will be valuable to clarify whether DAF-16 contributes to MYR-mediated longevity.

Rotenone-induced stress disrupted mitochondrial ultrastructure, reduced mitochondrial content, impaired respiratory chain complex I–III signals, decreased mitochondrial membrane potential, and lowered ATP levels [34]. MYR substantially reversed each of these changes. It also increased HSP-6 expression, consistent with activation of the mitochondrial unfolded protein response [35]. These observations prompted us to ask whether MYR selectively engages an MQC transcriptional program. Targeted qRT-PCR analysis of selected MQC-related genes showed that myricitrin (MYR) selectively upregulated *sir-2.1* [36,37,38], *atfs-1* [39], *hsp-6* [35], and *fzo-1* [40], and downregulated the fission gene *drp-1* [40]. This expression profile points to a coordinated mitochondrial quality control (MQC) response associated with UPR^mt^, mitochondrial fusion, and restrained fission [41]. Notably, SIR-2.1 represents a potential molecular connection: the aglycone myricetin activates the deacetylase SIRT1 (SIR-2.1 in *C. elegans*) to deacetylate PGC-1α and drive mitochondrial biogenesis [42], whereas dihydromyricetin, a myricetin derivative, promotes autophagic clearance of damaged mitochondria in *C. elegans* [23]. The upregulated *sir-2.1* mRNA levels observed herein support the involvement of SIR-2.1 in MYR-mediated mitochondrial regulation and lifespan extension. Future studies employing *sir-2.1* mutant strains will further clarify the mechanistic requirement of this gene for these beneficial phenotypes.

Taken together, our in vivo findings in *C. elegans* demonstrate that myricitrin (MYR) confers antioxidant protection by promoting DAF-16 nuclear translocation and regulating the transcriptional profile of mitochondrial quality control-related genes, thereby maintaining mitochondrial homeostasis. The elevated transcript level of *sir-2.1* suggests its potential involvement in this regulatory process, and its definitive causal role will be further verified by functional assays in subsequent studies.

Network pharmacology and molecular docking analyses further identified acetylcholinesterase as a candidate target. Following the cross-species network pharmacology approach described in the Section 2, human aging-related targets were mapped to their *C. elegans* orthologs. Computational modeling predicted favorable binding of MYR to the worm acetylcholinesterase homologs ACE-1 and ACE-2 (−9.3 and −11.0 kcal/mol, respectively). However, these human-to-worm computational projections remain predictive hypotheses. Given the role of cholinergic signaling in neuromuscular junction function and rhythmic movement [43,44], and consistent with the reported acetylcholinesterase-inhibitory activity of myricitrin in vitro (approximately 38% inhibition in a screening assay) [45], this computational interaction provides a plausible hypothesis for the observed improvements in motor performance. Molecular docking results have preliminarily suggested that MYR may improve motor function by targeting ACE-1/ACE-2. Future studies will combine biochemical enzyme activity assays with *ace-1*/*ace-2* genetic mutant experiments to clarify the functional involvement of this target at both enzymatic and genetic levels.

## 5. Conclusions

In summary, our study demonstrates that myricitrin (MYR) prolongs lifespan and improves locomotor function in *Caenorhabditis elegans* at an optimal concentration of 0.25 mg/mL without compromising growth, reproduction, or feeding, exhibiting a classic bell-shaped hormetic dose–response profile. Mechanistically, MYR exerts antioxidant effects by promoting DAF-16 nuclear translocation and maintaining mitochondrial homeostasis through the transcriptional regulation of mitochondrial quality control-related genes. Furthermore, cross-species network pharmacology and molecular docking analyses predict ACE-1 and ACE-2 as potential downstream targets underlying its motor-enhancing activity. Collectively, MYR represents a promising candidate compound for mitigating aging-related phenotypes, with its beneficial effects linked to a DAF-16-associated antioxidant response and SIR-2.1-correlated mitochondrial quality control network. Future studies will further elucidate the causal requirements of these key molecular targets and validate their anti-aging efficacy in higher organism models.

## Figures and Tables

**Figure 1:**
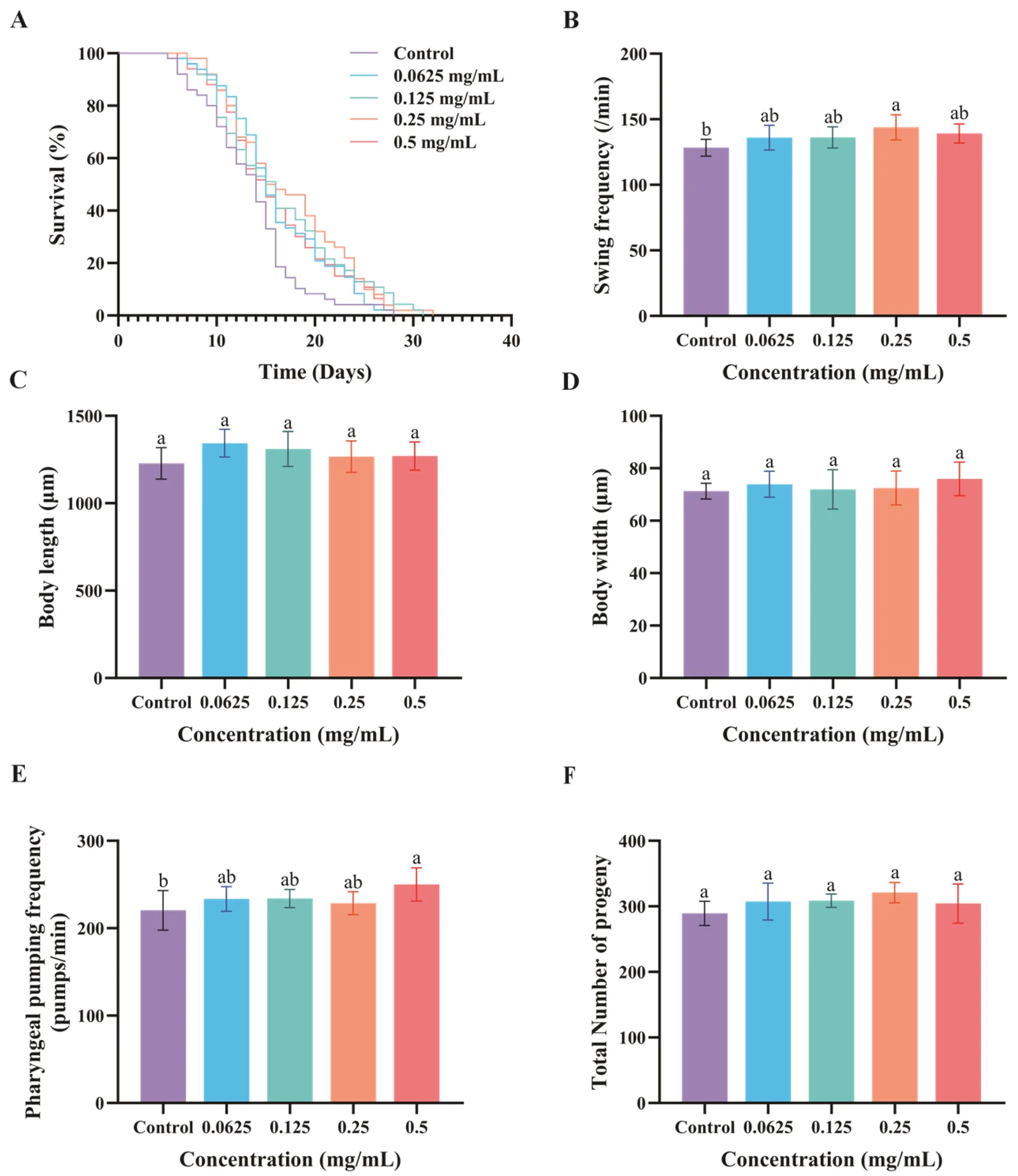
Myricitrin modulates physiological activities in *C. elegans*. (**A**) Survival curves of *C. elegans* following exposure to a range of MYR concentrations (0.0625–0.5 mg/mL) (N2, *n* = 50). (**B**) Quantification of body thrashing frequency, a motility parameter, in MYR-treated worms (N2, *n* = 6). (**C**,**D**) Analysis of morphological parameters, body length (**C**) and body width (**D**), in response to MYR treatment (N2, *n* = 6). (**E**) Pharyngeal pumping rate, an indicator of feeding behavior, in worms exposed to MYR (N2, *n* = 6). (**F**) Total progeny production (brood size) of *C. elegans* following MYR administration (N2, *n* = 3). Data are expressed as mean ± SD. Experiments were performed in three independent replicates. Bars labeled with different lowercase letters are statistically significantly different (*p* < 0.05, one-way ANOVA with LSD post hoc test).

**Figure 2:**
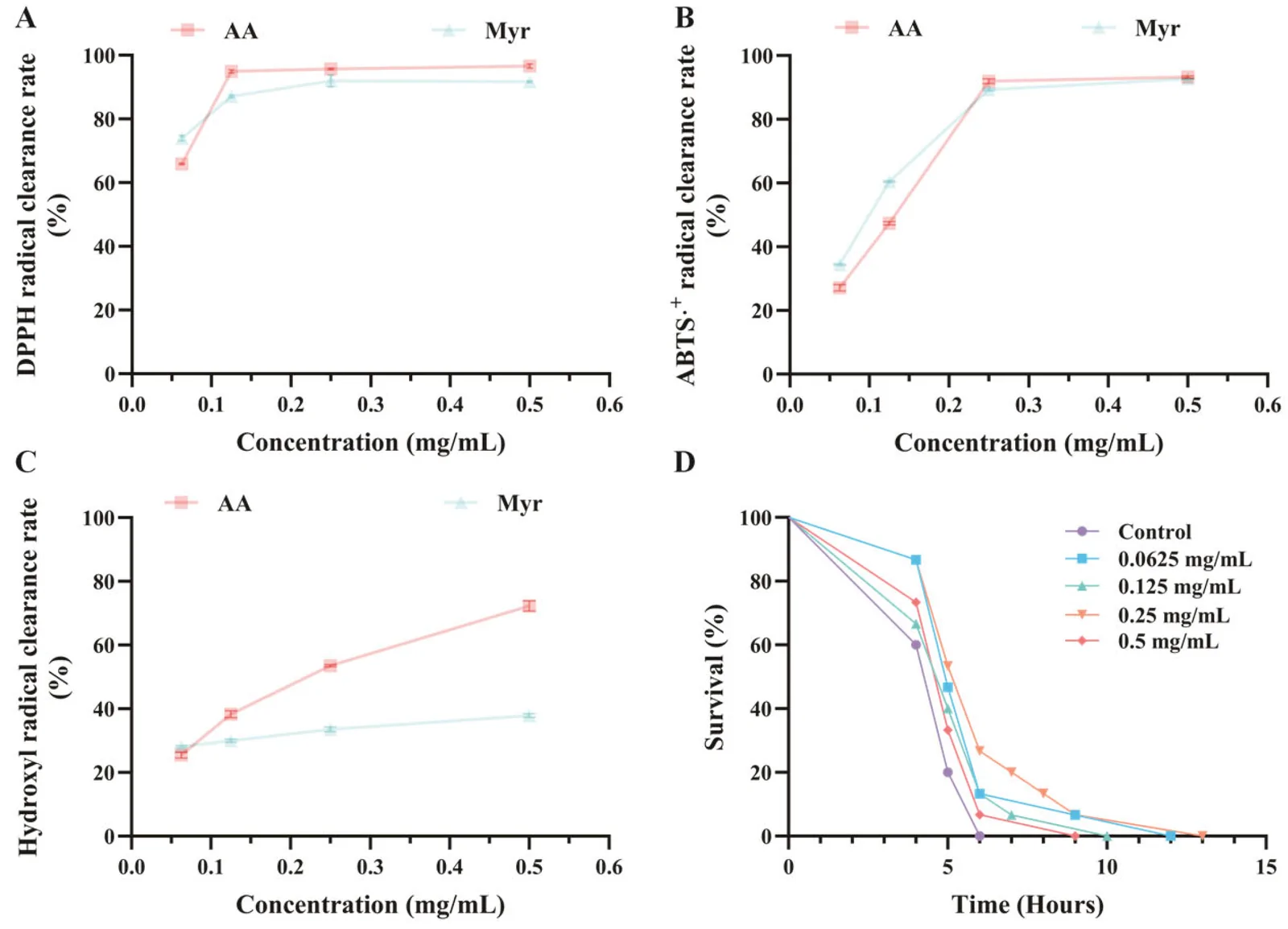
In vitro and in vivo antioxidant activity of myricitrin. (**A**–**C**) In vitro antioxidant activity of myricitrin (MYR) and ascorbic acid (AA). (**A**) Scavenging rate of DPPH free radicals. (**B**) Scavenging rate of ABTS^·+^ free radicals. (**C**) Scavenging rate of hydroxyl radicals (·OH). (**D**) Survival curves of MYR-treated (0.0625–0.5 mg/mL) *C. elegans* under oxidative stress (N2, *n* = 15). Data are expressed as mean ± SD. Experiments were performed in three independent replicates.

**Figure 3:**
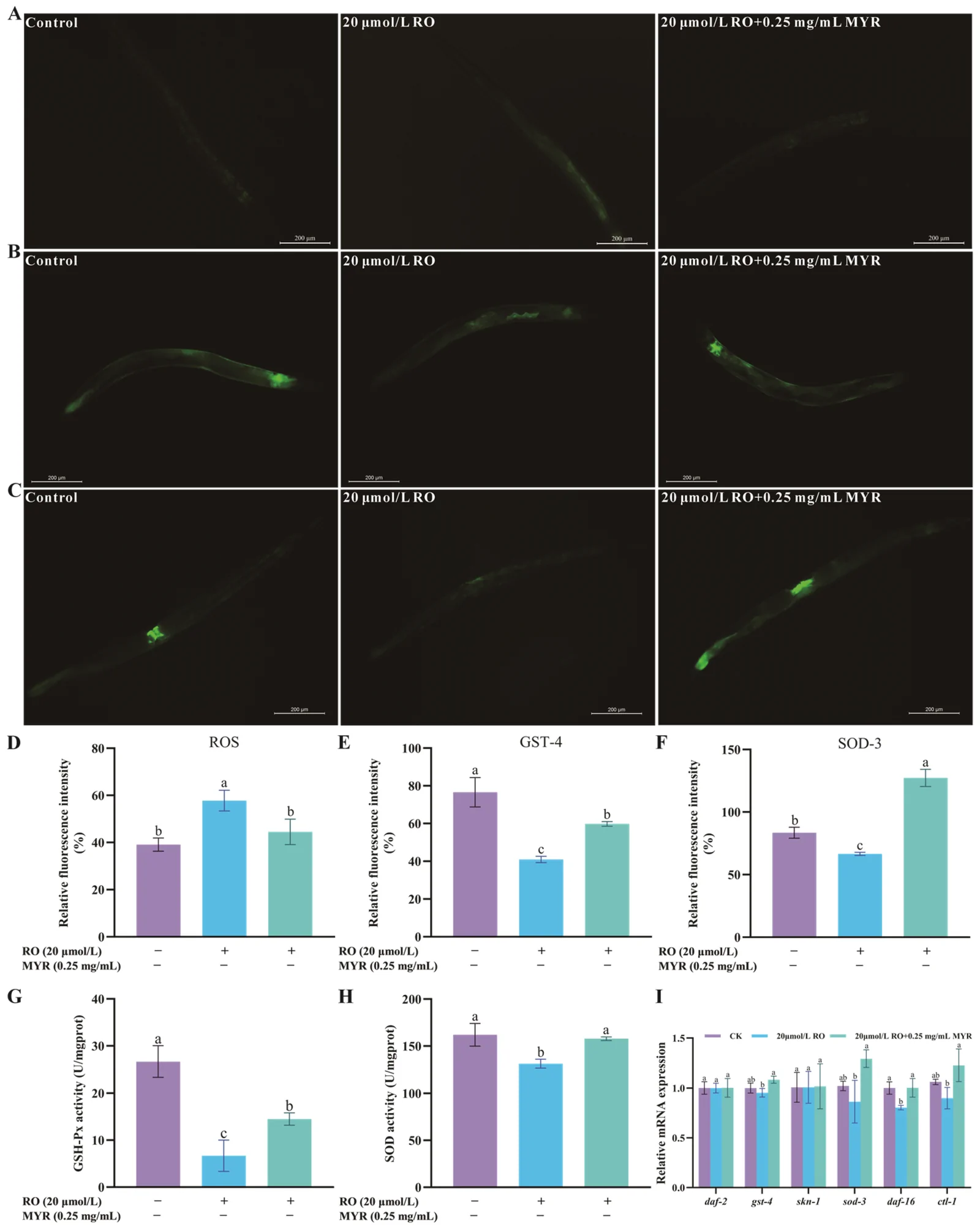
Effects of myricitrin on rotenone-induced oxidative damage. (**A**–**C**) Representative fluorescence micrographs of *C. elegans* ((**A**) N2. (**B**) CL2166. (**C**) CF1553). Magnification: 10×, scale bars: 200 μm. (**D**) Quantification of relative ROS fluorescence intensity in *C. elegans* (N2, *n* ≥ 6). (**E**,**F**) Quantification of relative GST-4 and SOD-3 fluorescence intensity in *C. elegans* (CL2166 for GST-4, *n* ≥ 6; CF1553 for SOD-3, *n* ≥ 6). (**G**,**H**) Relative enzymatic activities of GSH-Px and SOD in *C. elegans* (N2, *n* ≥ 50). (**I**) Relative mRNA expression levels of daf-2 and its downstream antioxidant genes (*daf-16*, *skn-1*, *gst-4*, *sod-3*, *ctl-1*) in *C. elegans* (N2, *n* ≥ 50). Data are expressed as mean ± SD. Experiments were performed in three independent replicates. Bars labeled with different lowercase letters are statistically significantly different (*p* < 0.05). The symbols “+” and “−“ denote treatment with or without the indicated agent, respectively.

**Figure 4:**
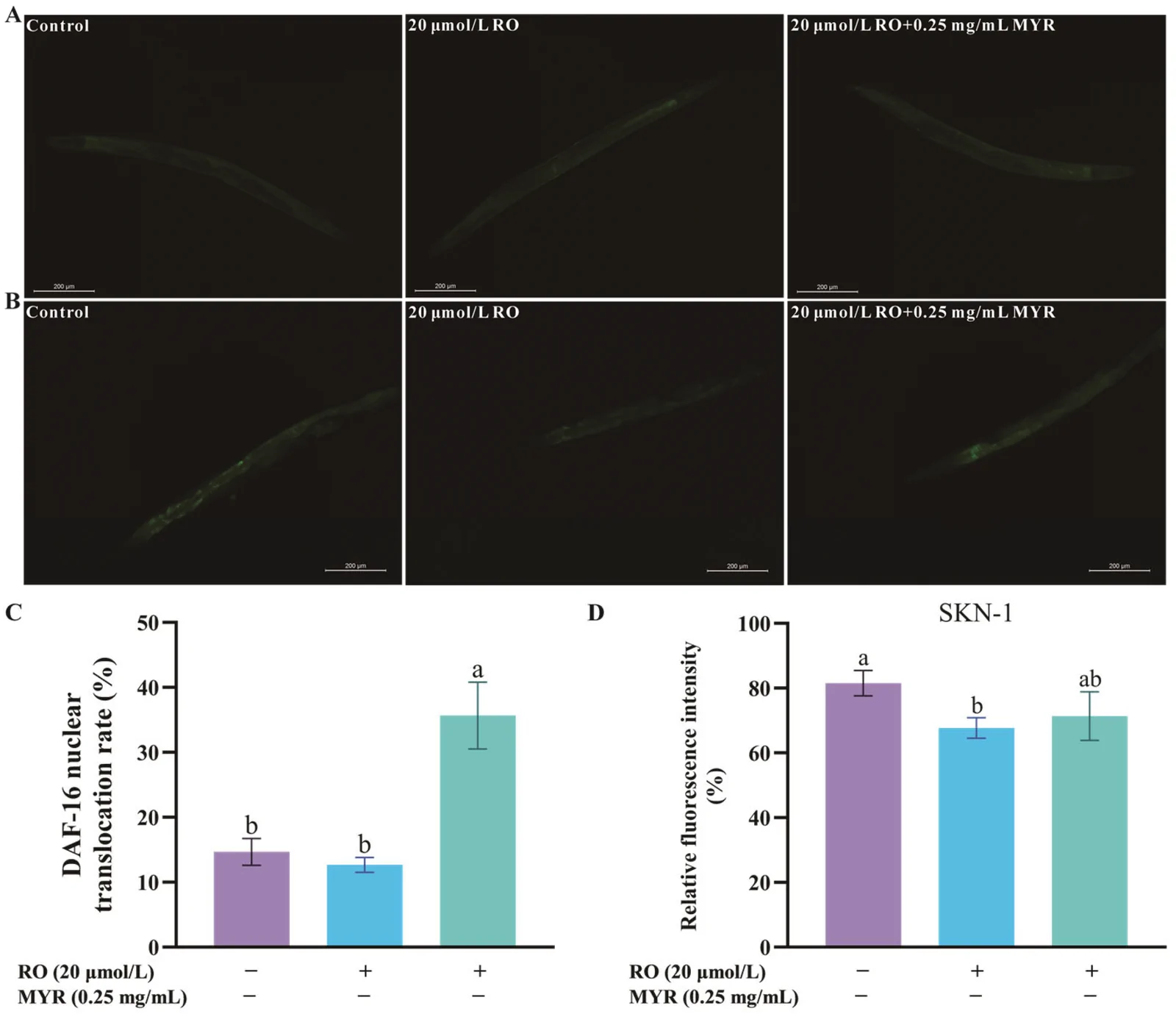
Effects of myricitrin on the expression and localization of the transcription factors DAF-16 and SKN-1. (**A**) Representative fluorescence micrographs of DAF-16::GFP localization (TJ356). Magnification: 10×, scale bars: 200 μm. (**B**) Representative fluorescence micrographs of SKN-1::GFP localization (LD1). Magnification: 10×, scale bars: 200 μm. (**C**) Quantification of the relative nuclear translocation of DAF-16 in *C. elegans* (TJ356, *n* = 6). (**D**) Quantification of the relative SKN-1::GFP fluorescence intensity in *C. elegans* (LD1, *n* = 6). Data are expressed as mean ± SD. Experiments were performed in three independent replicates. Bars labeled with different lowercase letters are statistically significantly different (*p* < 0.05). The symbols “+” and “−“ denote treatment with or without the indicated agent, respectively.

**Figure 5:**
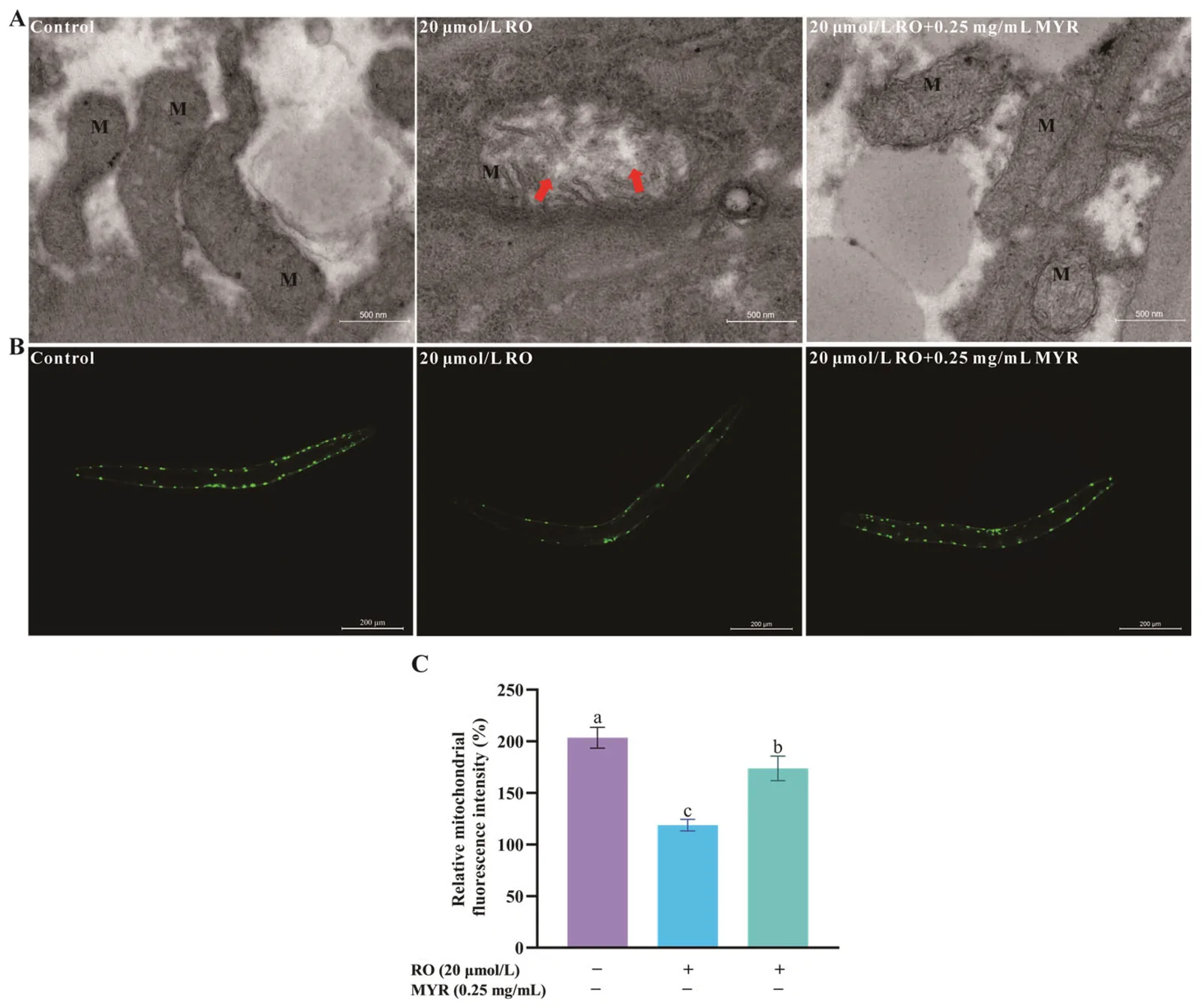
Myricitrin ameliorates rotenone-induced mitochondrial damage in *C. elegans*. (**A**) Representative transmission electron micrographs showing mitochondrial ultrastructure in *C. elegans* (PD4251). Original magnification: 20,000×; scale bars: 500 nm. (**B**) Representative fluorescence micrographs of the transgenic strain PD4251, visualizing mitochondria in body wall muscles (PD4251). Magnification: 10×, scale bars: 200 μm. (**C**) Quantification of relative mitochondrial fluorescence intensity in the PD4251 strain (PD4251, *n* = 6). Data are expressed as mean ± SD. Experiments were performed in three independent replicates. Bars labeled with different lowercase letters are statistically significantly different (*p* < 0.05). The symbols “+” and “−“ denote treatment with or without the indicated agent, respectively. In panel (**A**), the letter M marks mitochondria, and red arrows point to damaged internal mitochondrial structures.

**Figure 6:**
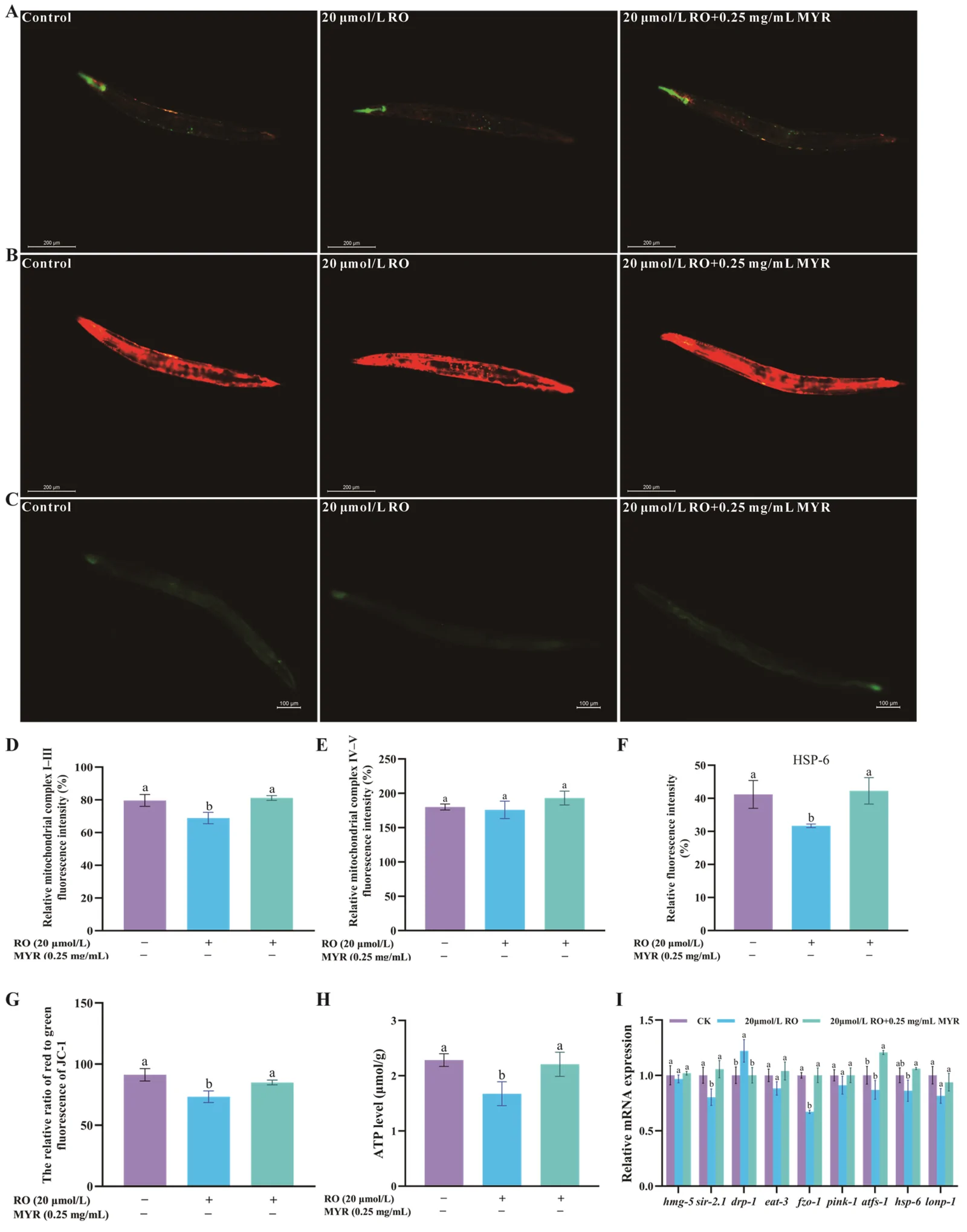
Myricitrin ameliorates rotenone-induced mitochondrial dysfunction in *C. elegans*. (**A**) Representative fluorescence micrographs of GFP-tagged mitochondrial complexes I–III in the transgenic strain CB7272. Magnification: 10×, scale bars: 200 μm. (**B**) Representative fluorescence micrographs of RFP-tagged mitochondrial complexes IV–V in the transgenic strain CB7272. Magnification: 10×, scale bars: 200 μm. (**C**) Representative fluorescence micrographs of HSP-6::GFP expression, a reporter for UPRmt activation, in the transgenic strain SJ4100. Magnification: 10×, scale bars: 200 μm. (**D**) Quantification of relative fluorescence intensity of GFP-tagged complexes I–III in strain CB7272 (*n* = 6). (**E**) Quantification of relative fluorescence intensity of RFP-tagged complexes IV–V in strain CB7272 (*n* = 6). (**F**) Quantification of relative HSP-6::GFP fluorescence intensity in strain SJ4100 (*n* = 6). (**G**) Quantification of relative mitochondrial membrane potential (ΔΨm) assessed by JC-1 staining (N2, *n* ≥ 6). (**H**) Quantification of cellular ATP content (N2, *n* = 6). (**I**) Relative mRNA expression levels of genes involved in mitochondrial quality control (N2, *n* ≥ 50). Data are expressed as mean ± SD. Experiments were performed in three independent replicates. Bars labeled with different lowercase letters are statistically significantly different (*p* < 0.05). The symbols “+” and “−“ denote treatment with or without the indicated agent, respectively.

**Figure 7:**
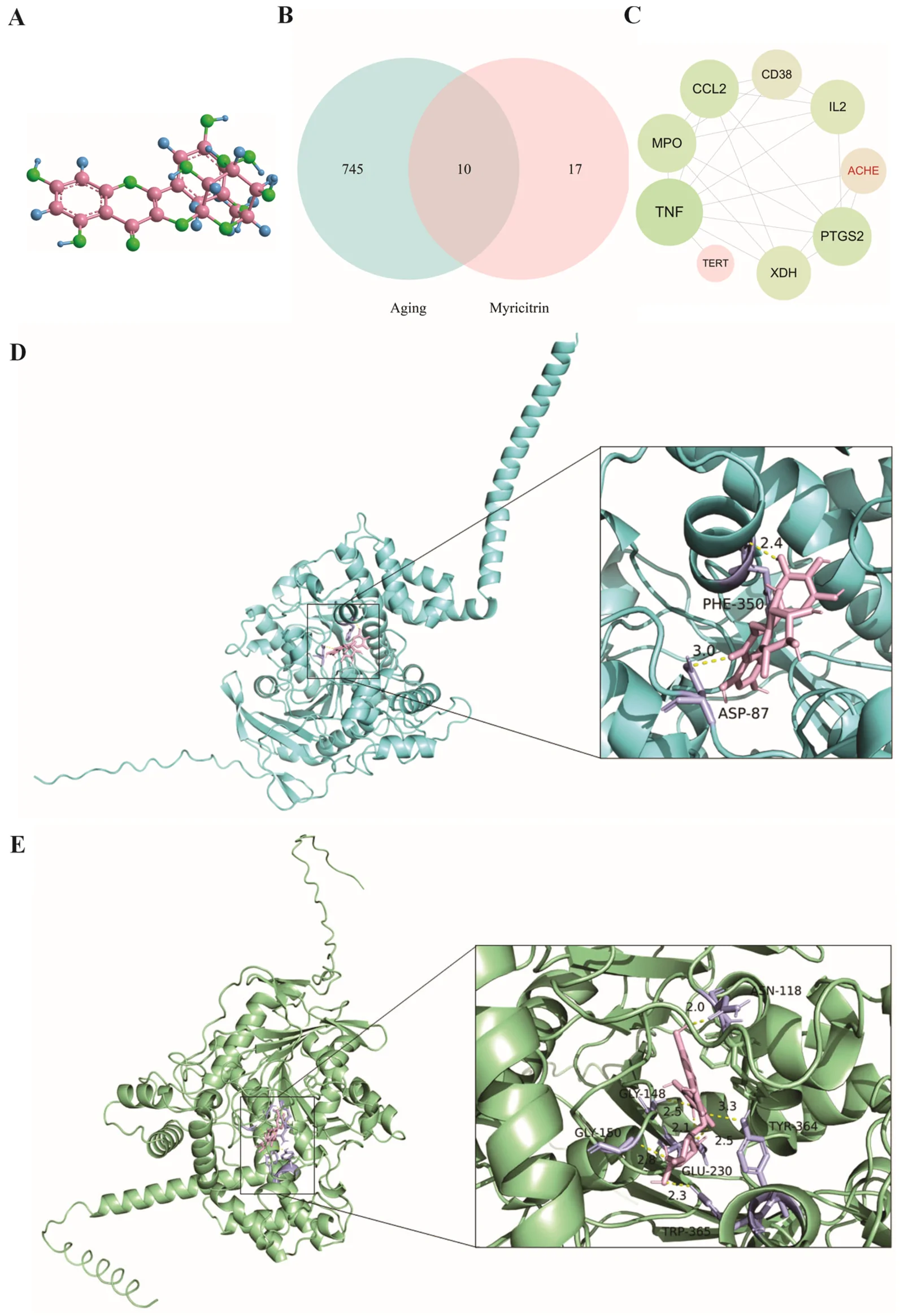
Putative targets of myricitrin (MYR) identified by network pharmacology and molecular docking. (**A**) Chemical structure of myricitrin (MYR). (**B**) Venn diagram showing the overlap between computationally predicted MYR-binding targets (pink) and human aging-associated proteins (green). The overlapping region indicates shared targets. (**C**) Protein–protein interaction (PPI) network of the overlapping target proteins. Node color intensity is scaled according to degree centrality, representing the number of interactions. (**D**) Predicted binding mode of MYR (pink sticks) within the active site of the *C. elegans* ACE-1 protein (cyan cartoon). Key hydrogen bonds are shown as yellow dashed lines. (**E**) Predicted binding mode of MYR (pink sticks) within the binding pocket of the *C. elegans* ACE-2 protein (green cartoon). Key hydrogen bonds are depicted as yellow dashed lines.

## Data Availability

The data will be made available upon request.

## Supplementary Materials

The following supporting information can be downloaded at https://www.mdpi.com/article/10.3390/antiox15101230/s1, Figure S1: The daily egg production of *C. elegans* following MYR administration; Figure S2: Effect of rotenone on the survival rate of *C. elegans*; Table S1: Strains, genotypes, and experimental purposes of *C. elegans* used in this study; Table S2: Primer sequences; Table S3: Effect of MYR on the lifespan of *C. elegans*; Table S4: Effect of MYR on the lifespan of *C. elegans* under oxidative stress conditions; Table S5: Potential targets of MYR.
