# Supplementary material for: Myricitrin Delays Caenorhabditis elegans Aging: Association with DAF-16, SIR-2.1, and Potential Cholinergic Modulation
Source: Antioxidants (Basel). 2026 Sep 23;15(10):1230. doi: 10.3390/antiox15101230 (PMC13700646; doi:10.3390/antiox15101230)
Supplement: Supplementary file 1 [file antioxidants-15-01230-s001.zip › antioxidants-4500997-supplementary.pdf]

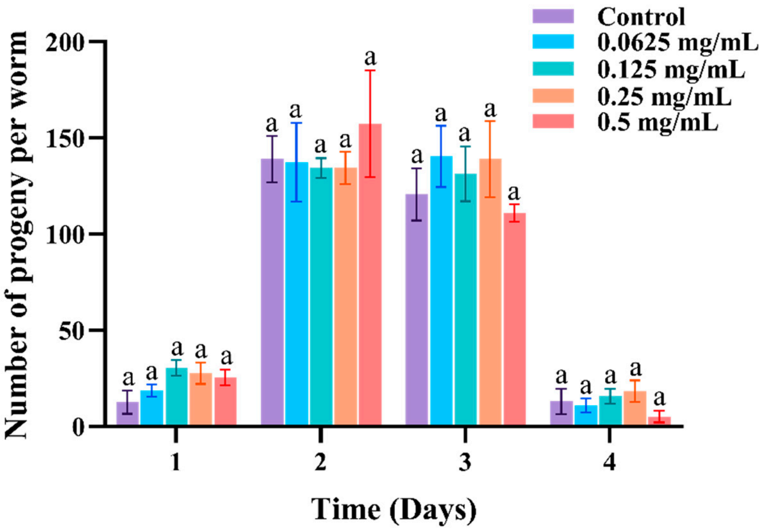

Figure S1. The daily egg production of *C. elegans* following MYR administration

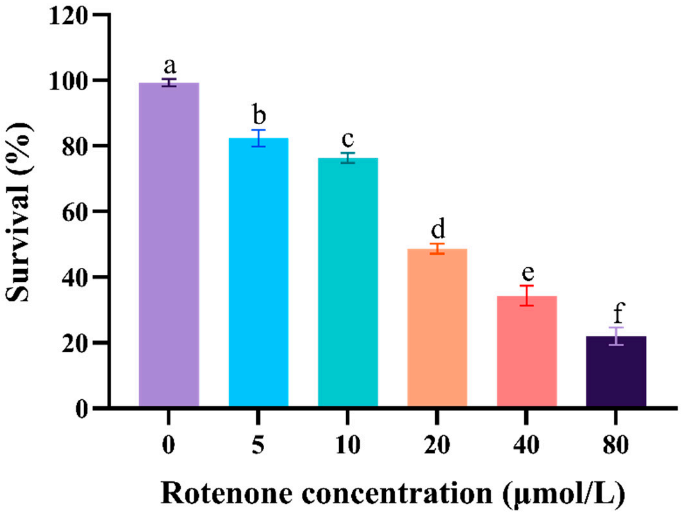

Figure S2. Effect of rotenone on the survival rate of *C. elegans*

Table S1. Strains, genotypes, and experimental purposes of *C. elegans* used in this study

C. elegans	Genotype	Source	Purpose
N2	Wild-type *C. elegans*	Kindly provided by Professor Wang Yuan from the State Key Laboratory of Biotherapy, Sichuan University.	serves as the standard wild-type reference
CL2166	*dvIs19 [(pAF15)gst-4p::GFP::NLS] III*		monitor GST-4::GFP fluorescence
LD1	*ldIs7 [skn-1b/c::GFP + rol-6(su1006)]*		evaluate SKN-1 nuclear translocation
CF1553	*muIs84 [(pAD76) sod-3p::GFP + rol-6(su1006)]*	Kindly provided by Professor Shan Ge from the University of Science	monitor SOD-3::GFP fluorescence

TJ356	*zIs356 [daf-16p::daf-16a/b::GFP + rol-6(su1006)]*	and Technology of China.	evaluate DAF-16 nuclear translocation
PD4251	*ccIs4251 [(pSAK2) myo-3p::GFP::LacZ::NLS + (pSAK4) myo-3p::mitochondrial GFP + dpy-20(+)] I*	Kindly provided by Jiangnan University.	assess mitochondrial content in body wall muscle cells
CB7272	*mIs12 [myo-2p::GFP + pes-10p::GFP + F22B7.9p::GFP] II. frIs7 [nlp-29p::GFP + col-12p::DsRed] IV. uIs69 [pCFJ90(myo-2p::mCherry) + unc-119p::sid-1] V*	Purchased from Sun Yat-sen University.	assess the activities of mitochondrial respiratory chain complexes I-V
SJ4100	*zcIs13 [hsp-6p::GFP + lin-15(+)]*	Purchased from Northwest A&F University.	evaluate the expression of the UPR^{mt} chaperone HSP-6

Table S2. Primer sequences

Primer name	Primer sequence (5'-3')
act-1	F: GCTGGACGTGATCTTACTGATTACC<br>R: GTAGCAGAGCTTCTCCTTGATGTC
sod-3	F: TTGAAGATCGCCACCTGTGCAAAC<br>R: ATGGACATAGTCTGGGCGGACATT
daf-16	F: AGGAGTCGAAGCCGATTAAGAC<br>R: GGTAGTGGCATTGGCTTGAAG
ctl-1	F: TGTCGTTCATGCCAAGGGAG<br>R: GATCCCGATTCTCCAGCGAC
gst-4	F: TGCAATGCTCAATGTGCCTT<br>R: TCAGCCCAAGTCAATGAGTCT
skn-1	F: GGACGTCAACAGCAGACTCA<br>R: TCTTGTCGTGACGATCCGTG
daf-2	F: ATCCCGGTCCGATTTCTT<br>R: GTTGATAATGCTGCCGAG
hmg-5	F: CAAGTAGACGCCACGCAAAG<br>R: CGCTTCGTCTGTGTACTTCTTTT
sir-2.1	F: TGTACTATCTGACGAGGATCGAA<br>R: CGCGTTTTTCAATGGGATTTGG
drp-1	F: AGAAAGAGCGGTCAGTGCT<br>R: GTTTCGAATAAGCCGTTCGAT
eat-3	F: GCGGCTAGAACGTGGTATGA<br>R: CGGACATCAATGAGGCGAGA
fzo-1	F: TGTTGCTTTCTGCAGGTTGA<br>R: AGTCGGCATTCCCCTGATTC
pink-1	F: GCTCTACGAATTGCTCGCCT<br>R: CTGCATTGCATCCTTGACCG

atfs-1	F: AAAAACGTGACGCTGGAAGC<br>R: TGGTGGCATTCCGTACTCATC
hsp-6	F: ACGCTGGAGATAAGATCATCG<br>R: AAGTTCGCACTTGGCCTTCT
lonp-1	F: GTAACACCGCCTGGAGTGAT<br>R: GGTCCGTCTTTTGGTGTTGC

Table S3. Effect of MYR on the lifespan of *C. elegans*

Concentration (mg/mL)	Mean lifespan (d)	Maximum lifespan (d)	*P* value
Control	13.53±0.71	28	
0.0625	16.14±0.77	28	0.0424
0.125	16.34±0.95	31	0.0139
0.25	17.26±0.88	32	0.0033
0.5	15.9±0.85	28	0.0629

* Data for mean lifespan are presented as mean ± SD. *P* values were calculated using the log-rank (Mantel-Cox) test compared to the Control group to assess differences in overall survival curves.

Table S4. Effect of MYR on the lifespan of *C. elegans* under oxidative stress conditions

Concentration (mg/mL)	Mean lifespan (h)	Maximum lifespan (h)	*P* value
Control	4.8±0.2	6	
0.0625	5.93±0.53	12	0.0413
0.125	5.4±0.41	10	0.19
0.25	6.27±0.6	13	0.0137
0.5	5.27±0.33	9	0.27

* Data for mean lifespan are presented as mean ± SD. *P* values were calculated using the log-rank (Mantel-Cox) test compared to the Control group to assess differences in overall survival curves.

Table S5 Potential targets of MYR

Database	Gene_symbol
TCMSP	MPO
SwissTargetPrediction	AKR1B1
	CA2
	CA7
	CA12
	CA4
	NOX4
	ADRA2C
	ACHE
	NQO2

	RPS6KA3
	NMUR2
	ADRA2A
	PTGS2
	CD38
	XDH
	TNF
	IL2
	PDE5A
	ADORA1
	ALOX5
	SLC29A1
	TERT
HERB	G6PC
	FASN
	GCK
	CCL2
	TNF
